# Laser Auriculotherapy for Anxiety Symptoms Across Diverse Populations: A Systematic Review and Meta-Analysis of Randomized Controlled Trials

**DOI:** 10.3390/ijerph23070919

**Published:** 2026-07-17

**Authors:** Hernán Andrés de la Barra Ortiz, Claudio Chamorro Lange, Nivaldo Antonio Parizotto, Richard Eloin Liebano

**Affiliations:** 1 Exercise and Rehabilitation Sciences Institute, School of Physical Therapy, Faculty of Rehabilitation Sciences, Universidad Andres Bello, Santiago 7591538, Chile; claudio.chamorro@unab.cl; 2 Institute of Advanced and Strategic Studies (IEAE), Federal University of São Carlos, São Carlos 13565-905, SP, Brazil; nivaldoaparizotto@hotmail.com; 3 Department of Rehabilitation Sciences, University of Hartford, West Hartford, CT 06117, USA; liebano@hartford.edu

**Keywords:** anxiety disorders, auriculotherapy, low-level laser therapy, photobiomodulation therapy, randomized controlled trials

## Abstract

**Highlights:**

**Public health relevance—How does this work relate to a public health issue?**

- Anxiety-related symptoms frequently coexist with chronic pain and musculoskeletal disorders, contributing to disability and reduced quality of life.
- Non-invasive interventions targeting psycho-emotional and autonomic regulation are increasingly relevant in contemporary rehabilitation and integrative healthcare.

**Public health significance—Why is this work of significance to public health?**

- This systematic review and meta-analysis suggests that LLLT-AT may reduce anxiety symptoms, pain intensity, and disability compared with control interventions, although these findings should be interpreted cautiously given the limited number of studies and the heterogeneity of the available evidence.
- LLLT-AT also showed a favorable safety profile, with lower risks of selected local adverse events compared with control interventions.

**Public health implications—What are the key implications or messages for practitioners, policy makers and/or researchers in public health?**

- LLLT-AT may represent a feasible, low-risk complementary intervention for managing anxiety-related symptoms across different clinical populations.
- Further high-quality randomized controlled trials with standardized dosimetry and longer follow-up are required before broad clinical implementation.

**Abstract:**

Anxiety disorders are highly prevalent conditions associated with substantial psychosocial burden, and low-level laser therapy auriculotherapy (LLLT-AT) has emerged as a non-invasive intervention with potential neuromodulatory effects for anxiety management. This systematic review aimed to evaluate the effects of LLLT-AT on anxiety symptoms across diverse populations. The review was conducted according to PRISMA 2020 guidelines and prospectively registered in PROSPERO (CRD420251159376). Randomized controlled trials comparing LLLT-AT with sham, placebo, or active interventions were identified through searches in PubMed, Web of Science, Scopus, CINAHL, MEDLINE, ScienceDirect, Cochrane Library, PEDro, and Google Scholar. The literature search was last updated on 2 July 2026. Risk of bias was assessed using RoB 2 and certainty of evidence using GRADE. Six RCTs involving 386 participants were included. Meta-analysis suggested significant reductions in post-treatment anxiety symptoms favoring LLLT-AT compared with control interventions (SMD = −0.65; 95% CI: −1.17 to −0.13), although substantial heterogeneity was identified (I^2^ = 78.9%). Significant reductions were also observed for pain intensity and disability, while adverse eventst analyses demonstrated lower risks of ear pain, hyperemia, and itchy ear following LLLT-AT. However, sensitivity analyses excluding studies with high risk of bias yielded a non-significant effect for anxiety outcomes (SMD = −0.35; 95% CI: −0.94 to 0.25). Interpretation of the findings should be considered cautiously due to substantial heterogeneity and the very low-to-low certainty of evidence for anxiety outcomes. Across all outcomes, the certainty of evidence ranged from very low to low, with very low certainty for anxiety outcomes and low certainty for pain intensity, disability, and adverse eventsoutcomes (ear pain, hyperemia, and itchy ear). LLLT-AT may represent a promising non-invasive complementary intervention for anxiety-related symptoms; however, these findings should be interpreted cautiously given the limited evidence base and substantial heterogeneity. Further high-quality randomized controlled trials with standardized protocols and longer follow-up assessments are required.

## 1. Introduction

Anxiety disorders are among the most prevalent mental health conditions worldwide and represent a major public health concern due to their chronic course and substantial psychosocial and functional burden [1,2]. Epidemiological evidence indicates that anxiety disorders affect approximately 301 million people globally and remain among the leading causes of years lived with disability [2]. Lifetime prevalence estimates may reach 33–34% in the general population, while prevalence in older adults has been estimated at 16.5% [1,2,3]. Anxiety disorders include conditions such as generalized anxiety disorder, panic disorder, social anxiety disorder, and agoraphobia, and are characterized by excessive fear, persistent worry, autonomic hyperarousal, and multiple somatic symptoms [4,5]. These disorders are strongly associated with sleep disturbances, fatigue, impaired occupational and social functioning, reduced quality of life, and increased healthcare utilization [2,5]. Furthermore, anxiety frequently coexists with chronic pain, depression, cardiovascular disease, cancer, and other chronic medical conditions, contributing to increased disability and socioeconomic burden worldwide [1,6].

Current management of anxiety disorders relies on both pharmacological and non-pharmacological strategies. Standard treatments typically combine cognitive-behavioral therapy with medications such as selective serotonin reuptake inhibitors, serotonin-norepinephrine reuptake inhibitors, and benzodiazepines [7,8]. Although these interventions can effectively alleviate symptoms, they still present notable drawbacks, including delayed onset of action, incomplete remission, adverse events, risk of relapse, limited accessibility, and poor adherence [4,8,9]. Consequently, interest has grown in complementary and integrative therapies that may relieve symptoms through minimally invasive, non-pharmacological approaches [10].

Auriculotherapy (AT) is a complementary therapeutic modality based on the stimulation of specific reflex points on the external ear that may influence physiological processes through neural and autonomic pathways [11]. Its theoretical framework integrates traditional Chinese medicine concepts and neurophysiological models proposing that the auricle contains a somatotopic representation of the human body [11]. The external ear is innervated by branches of the vagus, trigeminal, facial, and cervical nerves, which may provide anatomical pathways for interactions between auricular stimulation, autonomic regulation, and emotional processing [11,12,13].

Several techniques are used to stimulate auricular acupoints, including needles, seeds, electrical stimulation, and lasers [11,12]. Recent evidence suggests that non-invasive auricular interventions may improve patient acceptance and adherence due to their perceived comfort, flexibility, and minimal adverse events [14]. Among these, low-level laser therapy auriculotherapy (LLLT-AT) has gained attention as a non-invasive, painless, and aseptic approach that avoids skin penetration, potentially improving patient acceptance and adherence while allowing standardized dosimetry with minimal discomfort and adverse eventseffects [12,15,16,17].

LLLT, or photobiomodulation therapy, uses red or near-infrared light to stimulate auricular acupoints in a non-invasive manner. Similar to conventional auriculotherapy, LLLT-AT is thought to modulate neural and autonomic pathways through stimulation of the auricular branches of the vagus, trigeminal, facial, and cervical nerves, thereby influencing central nervous system structures involved in stress and emotional regulation [11,12]. In addition, photobiomodulation may exert neuromodulatory and anti-inflammatory effects that could further contribute to symptom improvement in anxiety-related conditions [18,19,20]. LLLT-AT has also demonstrated clinical applicability, a favorable safety profile, and a non-invasive nature [12,16,17].

Recent studies have examined LLLT-AT for emotional and psychological disorders. Reported benefits include reductions in anxiety, stress, and fatigue, as well as improvements in sleep quality and emotional well-being across different populations [21,22]. These findings are further supported by broader photobiomodulation research suggesting therapeutic potential in mood disorders such as anxiety and depression, possibly through the modulation of neural pathways involved in emotional regulation [23].

Despite these encouraging findings, important gaps remain regarding the effectiveness of LLLT-AT for anxiety symptoms. Previous systematic reviews have evaluated the effects of conventional auriculotherapy for a range of health conditions; however, these reviews primarily examined needle-, seed-, acupressure-, or electric-based interventions rather than laser auriculotherapy [11]. More recently, LLLT-AT has shown promising results for musculoskeletal pain [17], yet its effectiveness for anxiety symptoms has not been comprehensively synthesized. In addition, preliminary evidence suggests considerable variability in laser parameters, stimulated acupoints, intervention protocols, treatment duration, and outcome measures, which may limit comparability across studies [12,17,24,25]. Therefore, this systematic review aimed to evaluate the effects of LLLT-AT on anxiety symptoms across diverse populations. The primary outcome was anxiety symptom reduction, whereas pain intensity, disability, and adverse events were evaluated as secondary outcomes.

## 2. Materials and Methods

### 2.1. Study Design

This systematic review was conducted in accordance with the Preferred Reporting Items for Systematic Reviews and Meta-Analyses (PRISMA 2020) guidelines and was prospectively registered in the International Prospective Register of Systematic Reviews (PROSPERO; CRD420251159376). The review protocol was registered on 1 October 2025, before study selection and data extraction commenced [26,27].

### 2.2. Eligibility Criteria

The research question and eligibility criteria were developed according to the PICOS (Population, Intervention, Comparator, Outcomes, and Study Design) framework [28].

Eligible studies were randomized controlled trials (RCTs; parallel or crossover design) enrolling adults (≥18 years) from clinical or non-clinical populations in whom anxiety symptoms were assessed using validated anxiety assessment instruments, such as the State–Trait Anxiety Inventory (STAI), Beck Anxiety Inventory (BAI), Generalized Anxiety Disorder-7 (GAD-7), or Numeric Rating Scale for Anxiety (NRS-Anxiety). Interventions included LLLT-AT or auricular photobiomodulation therapy, administered either alone or in combination with other interventions, provided that identical co-interventions were applied across study groups and that LLLT-AT (or its placebo/sham equivalent) represented the principal between-group difference, thereby allowing the specific effect of the laser intervention to be interpreted. Comparators included sham/placebo interventions, no treatment, conventional treatments, or other active therapies.

The primary outcome was anxiety symptom reduction. Secondary outcomes included pain intensity assessed using validated instruments such as the Visual Analog Scale (VAS) and Numeric Rating Scale (NRS), disability, adverse events, and other related symptoms.

Studies that did not assess anxiety symptoms as an outcome were excluded, even if LLLT-AT was used as an intervention. Studies with incomplete data, unavailable full texts, or insufficient methodological information after attempts to contact the corresponding author were also excluded. No language restrictions were applied.

### 2.3. Search Strategy

A comprehensive electronic search was conducted in PubMed, Web of Science, Scopus, Cumulative Index to Nursing and Allied Health Literature (CINAHL), MEDLINE, ScienceDirect, and the Cochrane Library from inception, with the final literature search update performed on 2 July 2026. Additional manual searches were performed in the Physiotherapy Evidence Database (PEDro) and Google Scholar to identify potentially relevant studies. The database selection was based on recommendations to maximize literature coverage and retrieval sensitivity in systematic reviews [29].

The search strategy combined MeSH and free-text terms related to low-level laser therapy, photobiomodulation, auriculotherapy, ear acupuncture, and anxiety disorders using the Boolean operators “AND” and “OR”. The main search terms included “*Laser Therapy*”, “*Low-Level Light Therapy*”, “*Photobiomodulation*”, “*Photobiomodulation Therapy*”, “*Auriculotherapy*”, “*Acupuncture, Ear*”, “*Anxiety*”, “*Anxiety Disorders*”, “*Generalized Anxiety Disorder*” *and* “*Anxiety Symptoms*”. No search filters or restrictions were applied to maximize the identification of potentially relevant studies. The complete search strategy for each database is provided in Supplementary Table S1.

Additionally, the reference lists of eligible studies, reviews, systematic reviews, and meta-analyses identified through the search strategy were manually screened to identify further relevant articles.

### 2.4. Selection Process and Data Extraction

All records identified through the search strategy were imported into the Rayyan web application to facilitate duplicate removal and study screening [30]. Titles and abstracts were independently screened by two reviewers (HDB and CCH) according to the predefined eligibility criteria, followed by full-text assessment of potentially relevant studies. Disagreements during the selection process were resolved through discussion and consensus with a senior reviewer (NAP).

Data extraction was independently performed by two reviewers (HDB and CCH) using a standardized Microsoft Excel form, and the extracted data were subsequently verified by a senior reviewer (NAP). Any discrepancies in the extracted data were resolved through discussion and consensus among the three reviewers. When required, corresponding authors were contacted to obtain missing or unclear information. Extracted information included bibliographic characteristics (first author, year of publication, and country); methodological quality (PEDro score) [31]; eligibility criteria; participant characteristics; intervention groups; number of treatment sessions; outcomes and assessment instruments; assessment time points; post-treatment results; study conclusions; adverseevents; and sources of funding. Detailed laser parameters were additionally collected, including laser model, wavelength, emission mode (continuous or pulsed), output power, mean power, spot size, number of treatment points, treatment protocol, energy density, total energy, and treatment duration.

When numerical data were available only in graphical format, values were extracted using WebPlotDigitizer software (version 4.6, Pacifica, CA, USA) [32]. Any uncertainties were resolved through discussion and consensus among the review team, and corresponding authors were contacted when additional information was required.

### 2.5. Risk of Bias and Certainty of Evidence

The risk of bias (RoB) of the included RCTs was assessed using the Cochrane Risk of Bias 2 (RoB 2) tool, which evaluates bias arising from the randomization process, deviations from intended interventions, missing outcome data, outcome measurement, and selective reporting [33]. Each study was classified as having low RoB, some concerns, or high RoB according to the RoB 2 algorithm. Overall RoB judgments followed the RoB 2 algorithm, whereby studies with at least one high-risk domain or multiple domains with some concerns were classified as high RoB [34].

RoB assessments were independently performed by three reviewers (HDB, CCH, and NAP) using the Cochrane Risk of Bias 2 (RoB 2) tool. Inter-rater agreement was quantified using Fleiss’ kappa statistic. Any discrepancies among reviewers were resolved through discussion until consensus was reached. RoB visualizations were generated using the Robvis tool (https://www.riskofbias.info/welcome/robvis-visualization-tool; accessed on 13 July 2026) [35].

The certainty of evidence was assessed using the Grading of Recommendations Assessment, Development and Evaluation (GRADE) approach, considering RoB, inconsistency, indirectness, and imprecision [36]. Certainty of evidence was downgraded when important methodological limitations, substantial heterogeneity according to the I^2^ statistic [37], indirectness of evidence, or imprecise confidence intervals were identified. The certainty of evidence was rated as high, moderate, low, or very low, and outcome importance was defined a priori according to GRADE guidance and clinical relevance [36,38]. Anxiety symptom reduction and adverse eventswere considered critical outcomes, while all remaining outcomes were classified as important according to GRADE guidance. Summary of Findings tables were generated using GRADEpro GDT GRADEpro GDT (https://gdt.gradepro.org/app/, accessed on 2 July 2026).

### 2.6. Statistical Analysis

Meta-analyses were conducted when data from at least two studies were available for a given outcome. Means, standard deviations (SDs), and sample sizes (N) were extracted for experimental and control groups at baseline and post-intervention. When variability was reported as standard error (SE), SDs were calculated by multiplying the SE by the square root of the sample size, in accordance with Cochrane recommendations [39,40]. Outcomes reported as medians and interquartile ranges (IQRs; Q1–Q3) were converted into means and SDs using the formulas proposed by Wan et al. (2014) to enable inclusion in the quantitative synthesis [41].

For anxiety-related outcomes assessed using different instruments (e.g., STAI, BAI, and GAD-7), pooled standardized mean differences (SMDs) with 95% confidence intervals (CIs) were calculated. Hedges’ g was used as the effect size estimator to correct for small-sample bias [42]. Effect size magnitude was interpreted according to the thresholds proposed by Swinton et al. (2022), with values of 0.15, 0.45, and 0.80 indicating small, moderate, and large effects, respectively [43]. For outcomes measured using equivalent scales, pooled mean differences (MDs) were calculated and interpreted according to scale-specific clinical relevance and available minimal clinically important differences (MCIDs). When multiple instruments assessed the same construct, the most frequently used instrument across studies was selected. Meta-analyses for continuous outcomes were performed using the inverse variance method, assigning greater weight to more precise estimates [39]. For dichotomous outcomes, including adverse events, pooled Mantel–Haenszel risk ratios (RRs) with 95% CIs were calculated. In accordance with the recommendations of the Cochrane Handbook for Systematic Reviews of Interventions, studies reporting zero events in both groups were retained in the descriptive summary but did not contribute to the pooled RR estimates because they provide no information on the relative treatment effect [39,40].

The quantitative synthesis included studies evaluating LLLT-AT either as a standalone intervention or in combination with other therapeutic modalities. For three-arm trials, whenever a study arm was shared across more than one pairwise comparison, its sample size was divided between the corresponding pairwise comparisons, while the original means and standard deviations were retained for continuous outcomes, in accordance with the recommendations of the Cochrane Handbook for Systematic Reviews of Interventions [39]. This approach avoided double-counting of participants while preserving all relevant comparisons.

Subgroup analyses were planned to explore potential sources of heterogeneity according to comparator type (e.g., no treatment, placebo, or active control interventions) and clinical population type (e.g., TMD-related anxiety, parental perioperative anxiety, university student anxiety, and breast cancer-related anxiety), when sufficient data were available.

Between-study heterogeneity was assessed using Cochran’s Q test and quantified using the tau-squared (τ^2^) estimator and the I^2^ statistic, with I^2^ values of approximately 25%, 50%, and 75% interpreted as low, moderate, and substantial heterogeneity, respectively [37,39]. Random-effects models using the restricted maximum likelihood (REML) estimator with Knapp–Hartung adjustment were used when moderate or substantial heterogeneity was identified, whereas inverse-variance fixed-effect models were considered when heterogeneity was negligible [44].

Sensitivity analyses were conducted to assess the robustness of pooled estimates. Publication bias was planned to be assessed using funnel plots and Egger’s regression test when at least 10 studies were available [45].

Statistical analyses were performed using R software (version 4.4.2; R Foundation for Statistical Computing, Vienna, Austria),with significance set at *p* ≤ 0.05.

### 2.7. Clinical Relevance

Clinical relevance was interpreted according to effect size magnitude and available scale-specific MCIDs. Hedges’ g values of 0.15, 0.45, and 0.80 were considered small, moderate, and large effects, respectively, according to Swinton et al. (2022) [43].

## 3. Results

### 3.1. Search Results

The systematic search identified 5410 records across PubMed, Scopus, Web of Science, Cumulative Index to Nursing and Allied Health Literature (CINAHL), MEDLINE, ScienceDirect, the Cochrane Library, and PEDro (last updated on 2 July 2026). Additional search methods identified a further 9610 records, primarily through Google Scholar. After duplicate removal, records were screened by title and abstract according to the predefined eligibility criteria. Following full-text assessment, six RCTs were considered eligible and included in the qualitative and quantitative synthesis [24,25,46,47,48,49].

**Figure 1:**
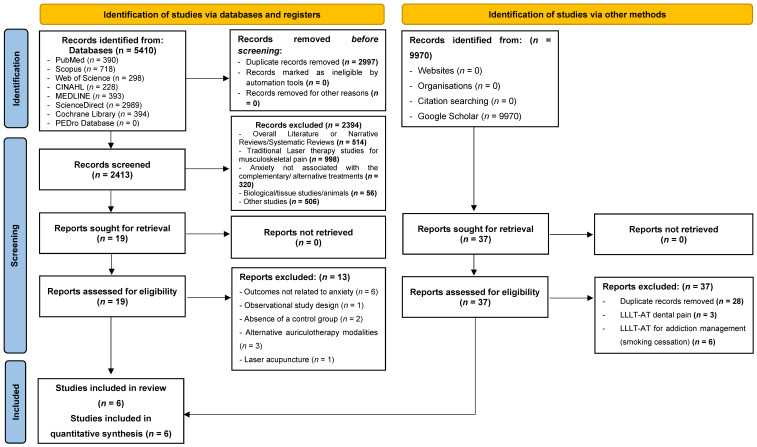
PRISMA flowchart.

Thirteen studies identified through database searching were excluded during full-text assessment for the following reasons: observational/cohort study design (*n* = 1), absence of a control group (*n* = 2), use of auriculotherapy modalities other than low-level laser therapy auriculotherapy (LLLT-AT) (*n* = 3), evaluation of outcomes not related to anxiety (*n* = 6), and investigation of laser acupuncture rather than auricular laser therapy (*n* = 1). Regarding studies identified through alternative search methods, 37 reports underwent full-text eligibility assessment. All were excluded due to duplicate records (*n* = 28), investigation of LLLT-AT for dental pain conditions (*n* = 3), or evaluation of LLLT-AT for addiction management, specifically smoking cessation (*n* = 6). Figure 1 presents the PRISMA flow diagram summarizing the study selection process, while Supplementary Table S1 details the complete search strategy and Supplementary Table S2 summarizes the excluded studies and reasons for exclusion.

### 3.2. RoB Assessment

**Figure 2:**
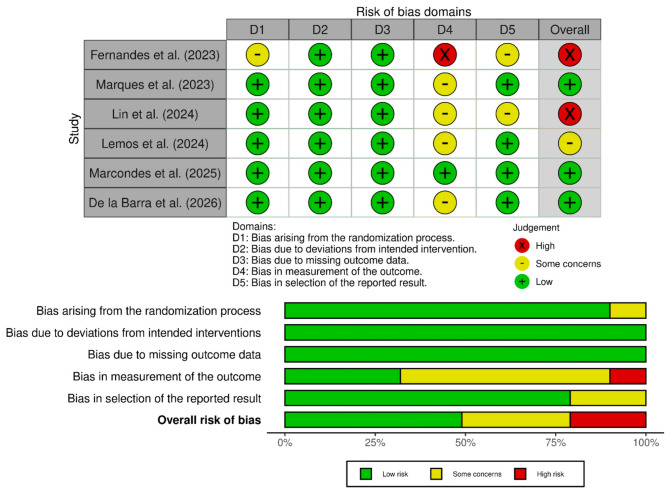
Overall and Domain-Specific Risk of Bias Assessment [24,25,46,47,48,49].

Figure 2 presents the RoB 2 assessment of the included RCTs [34]. The risk of bias assessments were independently performed by three reviewers (HDB, CCH, and NAP), and the final judgment for each study was reached by consensus. Across the six included studies, the average Fleiss’ κ prior to consensus was 0.75, indicating substantial agreement according to the Landis and Koch classification. Based on the overall RoB assessment, three studies were judged as having low RoB, one study as presenting some concerns, and two studies as having high RoB. The main methodological limitations were associated with bias in measurement of the outcome (D4) and bias in selection of the reported result (D5), whereas all studies demonstrated low RoB for deviations from intended interventions (D2) and missing outcome data (D3). Most trials also showed low RoB arising from the randomization process (D1), although one study presented some concerns regarding the randomization process. RoB plots were weighted according to each study’s inverse-variance contribution to the primary meta-analysis of anxiety outcomes [33,39].

### 3.3. Study Characteristics

Table 1 summarizes the characteristics of the included RCTs, which evaluated heterogeneous clinical populations, including individuals with temporomandibular disorders and anxiety [24,25,49], parental anxiety during children’s surgery [46], anxiety in university students [47], and women with advanced breast cancer undergoing chemotherapy [48].

Most of the included RCTs were conducted in Brazil (*n* = 4) [24,25,47,48], followed by one study from the USA [46] and one from Chile [49]. The PEDro scores of the included studies ranged from 5/10 to 9/10, with a mean score of 7.0 ± 1.4 [24,25,46,47,48,49], suggesting overall moderate-to-high methodological quality among the included RCTs, as PEDro scores ≥ 6 are generally considered indicative of good methodological quality [50]. The most frequently unmet PEDro criteria were therapist and participant blinding, whereas random allocation, baseline comparability, and between-group statistical comparisons were consistently satisfied across most studies. Detailed PEDro scores are presented in Supplementary Table S3.

A total of 386 participants were enrolled across the included RCTs, with 163 allocated to LLLT-AT-related interventions and 223 assigned to control conditions [24,25,46,47,48,49]. Among the studies reporting sex distribution, 269 participants were women and 52 were men, whereas two studies did not report sex [25,46]. Control interventions included no treatment [24], sham/placebo laser auriculotherapy [25,46,48,49], non-anxiolytic auricular acupoints [46], needle-based auricular acupuncture [47] and chemotherapy combined with multidisciplinary care [48]. Additionally, one study combined sham LLLT-AT with myofascial release as an active comparator [49].

The intervention frequency ranged from 1 to 2 sessions per week [24,25,46,47,48,49], with treatment durations varying from 1 to 10 weeks. The total number of sessions ranged from 1 to 10 across the included RCTs. Assessment time points varied across studies; however, all included a baseline assessment (T0) and at least one post-treatment evaluation [24,25,47,48]. Follow-up assessments were conducted in three studies, with evaluation periods ranging from 30 min post-treatment to 4 weeks after the intervention [46,47,49].

### 3.4. Assessment Procedures and Outcome Measures

Anxiety-related outcomes were primarily assessed using validated self-reported instruments, including the STAI [46,47,48], BAI [24], GAD-7 [25,49], NRS adapted for anxiety (NRS-anxiety) [46], and Patient Health Questionnaire-4 (PHQ-4) [25].

Pain-related outcomes included pain intensity assessed using the GCPS [25], pain-related symptoms derived from the QLQ-C30 [48], and mechanical pain sensitivity evaluated through pressure pain threshold (PPT) using pressure algometry [49]. Additional outcomes included sleep quality (SQ) [24], disability and mandibular functional limitation assessed using the Jaw Functional Limitation Scale-8 (JFLS-8) [25,49], mandibular movement measurements [25], maximum mouth opening range of motion [49], health-related quality of life (HRQoL), functionality, and fatigue [48], as well as physiological parameters such as heart and respiratory rate, treatment satisfaction, and perceived treatment acceptability [46,47].

### 3.5. Laser Parameters of Included Studies

The laser dosimetry and treatment parameters of the included RCTs are presented in Table 2. Laser parameters varied considerably across studies. Wavelengths ranged from 660 to 905 nm [24,25,46,47,48,49], with most studies using red or near-infrared spectra. Continuous emission mode was reported by Lemos et al. and Marcondes et al. [47,48], whereas pulsed emission was used by Marques et al. and De la Barra et al. [25,49]; Fernandes et al. and Lin et al. did not specify the emission mode [24,46]. Peak power ranged from 0.1 to 13.5 W [24,49], whereas energy density varied from 0.19 to 4 J/cm^2^ [25,46,48,49]. The number of auricular stimulation points ranged from 4 to 16, and total delivered energy varied between 9 J [46] and 64 J [25], with a mean total delivered energy of 28.6 ± 21.4 J. Treatment time ranged from 20 to 56 s per point, corresponding to total application times between 220 and 640 s [24,25,46,47,48,49]. Among the studies reporting total application time, the mean treatment duration was 340.8 ± 170.4 s [24,25,46,47,48,49].

**Figure 3:**
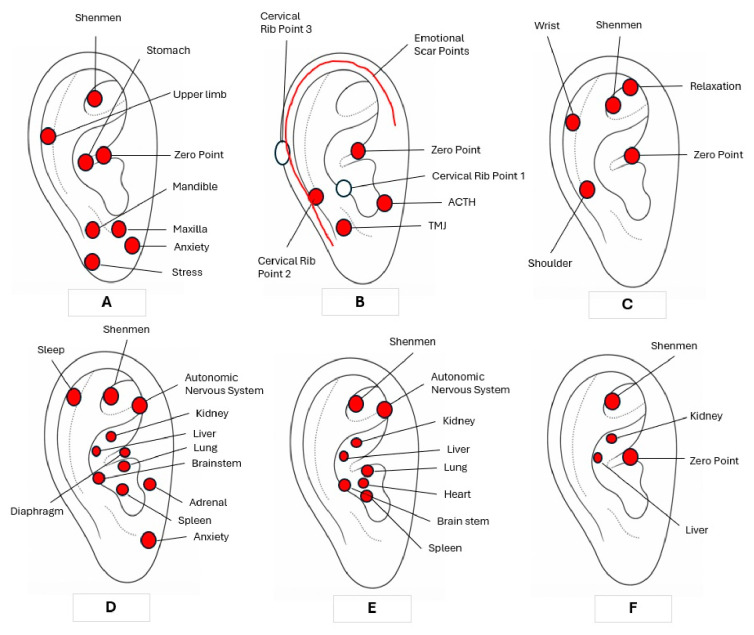
Auricular acupoint maps and treatment protocols used across the included studies: (**A**) Fernandes et al. (8 points); (**B**) Marques et al. (16 points); (**C**) Lin et al. (6 points); (**D**) Lemos et al. (11 points); (**E**) Marcondes et al. (8 points); and (**F**) De la Barra et al. (4 points). Red circles indicate externally visible auricular acupoints, whereas white circles represent internal or anatomically concealed auricular points used in the treatment protocols.

The included studies applied LLLT-AT protocols using between 4 and 16 auricular points per session. Across the included studies, the most commonly stimulated auricular points were Shenmen, Point Zero (O’), Kidney, Liver, and the Autonomic Nervous System [24,25,46,47,48,49]. Additional points targeted anatomical regions and symptom-related conditions, including the Brainstem, TMJ, Mandible, Maxilla, Shoulder, Wrist, Upper Limb, and points related to anxiety, stress, relaxation, and insomnia. The combinations and number of stimulated auricular points varied across the included protocols. Figure 3 illustrates the auricular acupoints applied across the included studies.

### 3.6. Meta-Analysis

**Figure 4:**
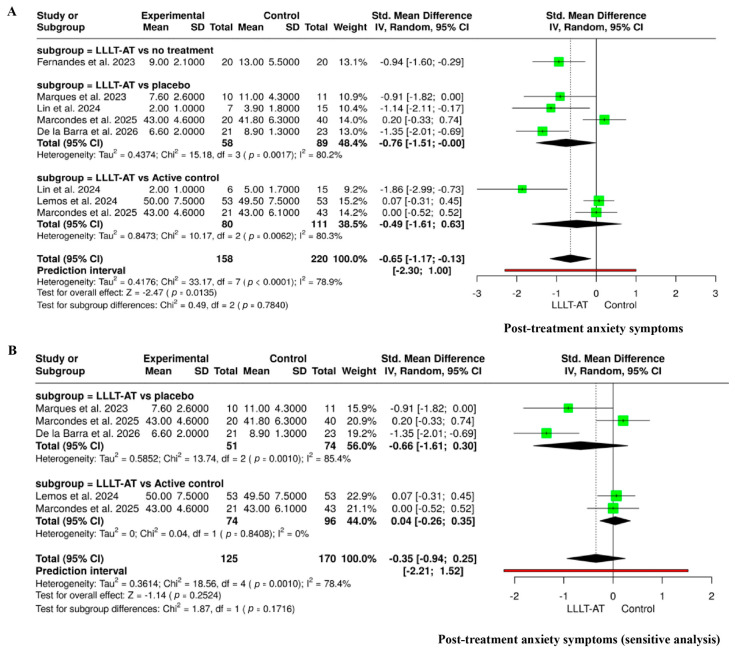
Forest plots illustrating the effects of LLLT-AT on post-treatment anxiety symptoms. (**A**) Primary meta-analysis including all eligible comparisons according to control condition (no treatment, placebo, and active control). (**B**) Sensitivity analysis excluding the studies by Fernandes et al. and Lin et al. because of RoB. Effect estimates are reported as SMDs with 95% confidence intervals derived from random-effects models. Negative SMD values indicate lower post-treatment anxiety scores in the LLLT-AT groups relative to control conditions [24,25,46,47,48,49].

*Anxiety outcome.* Figure 4A presents the meta-analysis of anxiety-related outcomes comparing LLLT-AT with control interventions. Six studies comprising eight comparisons were included in the quantitative synthesis, involving 158 participants in the LLLT-AT groups and 220 participants in the control groups. Anxiety symptoms were assessed using validated instruments, including the BAI [24], GAD-7 [25,49], NRS-Anxiety [46], and STAI-S [47]. No included studies required conversion of SEs or median/IQR data for quantitative synthesis.

Subgroup analyses were conducted according to comparator type, including no treatment, placebo, and active control interventions. Lin et al. and Marcondes et al. employed three-arm designs; therefore, split-comparison procedures were applied according to Cochrane recommendations to avoid double-counting participants [39]. Pooled analyses were performed using random-effects models with the inverse variance method, REML estimation, and Knapp–Hartung adjustment. Significant between-study heterogeneity was identified (Q = 33.17, df = 7, *p* < 0.001; τ^2^ = 0.42; τ = 0.65; H = 2.18; I^2^ = 78.9%) [37,40].

For subgroup analyses, LLLT-AT showed a significant effect compared with no treatment in one study (SMD = −0.94; 95% CI: −1.60 to −0.29). The pooled placebo-controlled subgroup also favored LLLT-AT (SMD = −0.76; 95% CI: −1.51 to −0.00), whereas no statistically significant difference was observed compared with active control interventions (SMD = −0.49; 95% CI: −1.61 to 0.63; *p* > 0.05). The test for subgroup differences was not significant (χ^2^ = 0.49, df = 2, *p* = 0.7840). Overall, LLLT-AT significantly reduced anxiety symptoms compared with control interventions (SMD = −0.65; 95% CI: −1.17 to −0.13; *p* < 0.05). Based on predefined Hedges’ g thresholds, this effect was interpreted as moderate and potentially clinically relevant [43].

Due to the substantial variability in follow-up assessment time points across studies, no quantitative synthesis was performed for follow-up anxiety outcomes [24,25,46,47,48,49].

*Anxiety outcome (sensitive analysis).* Figure 4B presents the sensitivity analysis for anxiety-related outcomes after excluding studies with high RoB [24,46]. Four studies comprising five comparisons were included, involving 125 participants in the experimental groups and 170 participants in the control groups. Pooled analyses were performed using random-effects models with the inverse variance method. Significant between-study heterogeneity was identified (Q = 18.56, df = 4, *p* = 0.0010; τ^2^ = 0.36; I^2^ = 78.4%). The pooled analysis demonstrated no statistically significant difference between LLLT-AT and control interventions (SMD = −0.35; 95% CI: −0.94 to 0.25; *p* = 0.2524). Compared with the primary analysis, the exclusion of studies with high RoB resulted in a reduced effect estimate, indicating that the overall anxiety findings should be interpreted with caution.

According to the GRADE assessment, the certainty of evidence for anxiety outcomes ranged from very low to low, reflecting concerns mainly related to risk of bias, inconsistency, indirectness, and imprecision across the available evidence (Table 3) [36].

**Figure 5:**
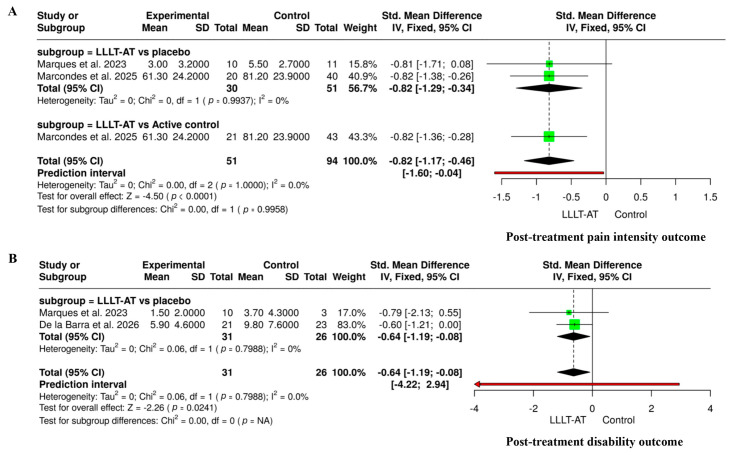
Forest plot for post-treatment outcomes following LLLT-AT interventions. (**A**) Comparison of post-treatment pain intensity between LLLT-AT and placebo, as well as between LLLT-AT and active control interventions. (**B**) Comparison of post-treatment disability between LLLT-AT and placebo controls. Negative SMD values favor LLLT-AT for reductions in pain intensity and disability [25,48,49].

*Pain intensity outcome.* Figure 5A presents the meta-analysis of pain intensity outcomes comparing LLLT-AT with placebo and active control interventions [25,48]. Three comparisons from two studies were included in the quantitative synthesis, involving 51 participants in the LLLT-AT groups and 94 participants in the control groups. Split-comparison procedures were applied for the three-arm study by Marcondes et al. according to Cochrane recommendations [39]. Pain intensity outcomes were assessed using the GCPS and the pain domain of the QLQ-C30. Pain intensity outcomes were pooled using SMDs with fixed-effect models and the inverse variance method. No between-study heterogeneity was identified (Q = 0.00, df = 2, *p* = 1.00; τ^2^ = 0.00; τ = 0.00; H = 1.00; I^2^ = 0.0%).

Subgroup analyses demonstrated a significant reduction in pain intensity favoring LLLT-AT compared with placebo interventions (SMD = −0.82; 95% CI: −1.29 to −0.34). Similarly, the comparison with active control interventions also favored LLLT-AT (SMD = −0.82; 95% CI: −1.36 to −0.28; *p* < 0.05). The test for subgroup differences was not significant (χ^2^ = 0.00, df = 1, *p* = 0.9958).

Overall, the pooled analysis demonstrated a statistically significant reduction in pain intensity favoring LLLT-AT compared with control interventions (SMD = −0.82; 95% CI: −1.17 to −0.46; *p* < 0.01). Based on the predefined Hedges’ g thresholds, the pooled effect size was interpreted as large, suggesting potential clinical relevance [43].

According to the GRADE assessment, the certainty of evidence for pain intensity outcomes was low, mainly due to concerns regarding risk of bias, indirectness, and imprecision (Table 3) [36].

*Disability outcome.* Figure 5B presents the meta-analysis of disability-related outcomes comparing LLLT-AT with placebo interventions. Two studies were included in the quantitative synthesis, involving 31 participants in the LLLT-AT groups and 26 participants in the control groups [25,49]. Disability outcomes were assessed using the JFLS-8. Given the absence of between-study heterogeneity, pooled analyses were performed using fixed-effect models with the inverse variance method.

The pooled analysis demonstrated a statistically significant reduction in disability favoring LLLT-AT compared with placebo interventions (SMD = −0.64; 95% CI: −1.19 to −0.08; *p* < 0.05). Based on the predefined Hedges’ g interpretation thresholds, the pooled effect size was interpreted as moderate, suggesting potential clinical relevance [43].

According to the GRADE assessment, the certainty of evidence for disability outcomes was classified as low (Table 3) [36].

**Figure 6:**
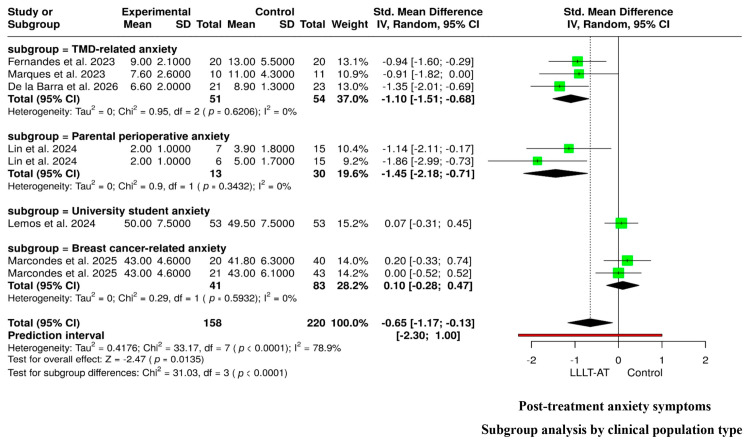
Forest plot of the exploratory subgroup analysis according to clinical population type for post-treatment anxiety outcomes following LLLT-AT interventions. Subgroups were defined as TMD-related anxiety, parental perioperative anxiety, university student anxiety, and breast cancer-related anxiety [24,25,46,47,48,49].

*Anxiety outcome (subgroup analysis according to clinical population type).* Figure 6 presents the exploratory subgroup analysis according to clinical population type for post-treatment anxiety outcomes. This analysis included six studies comprising eight comparisons and was performed to investigate potential sources of the substantial heterogeneity observed in the primary anxiety analysis (I^2^ = 78.9%). For the three-arm trials conducted by Lin et al. [46] and Marcondes et al. [48], split-comparison procedures were applied according to Cochrane recommendations by dividing the shared study arm between comparisons to avoid double-counting participants. Significant subgroup differences were identified (χ^2^ = 31.03, df = 3, *p* < 0.001). LLLT-AT showed significant effects in participants with TMD-related anxiety (SMD = −1.10; 95% CI: −1.51 to −0.68; I^2^ = 0%) and parental perioperative anxiety (SMD = −1.45; 95% CI: −2.18 to −0.71; I^2^ = 0%). Conversely, no significant effects were observed for university student anxiety (SMD = 0.07; 95% CI: −0.31 to 0.45) or breast cancer-related anxiety (SMD = 0.10; 95% CI: −0.28 to 0.47). Based on the predefined Hedges’ g thresholds, the effects observed for TMD-related anxiety and parental perioperative anxiety were classified as large, whereas those observed for university student anxiety and breast cancer-related anxiety were classified as negligible [43].

According to the GRADE assessment, the certainty of evidence was low for all subgroup analyses of anxiety outcomes, including TMD-related anxiety, parental perioperative anxiety, university student anxiety, and breast cancer-related anxiety (Table 3).

**Figure 7:**
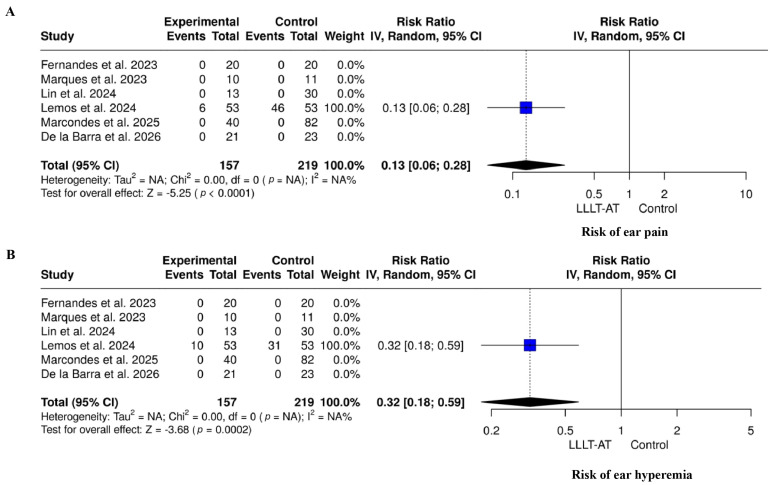

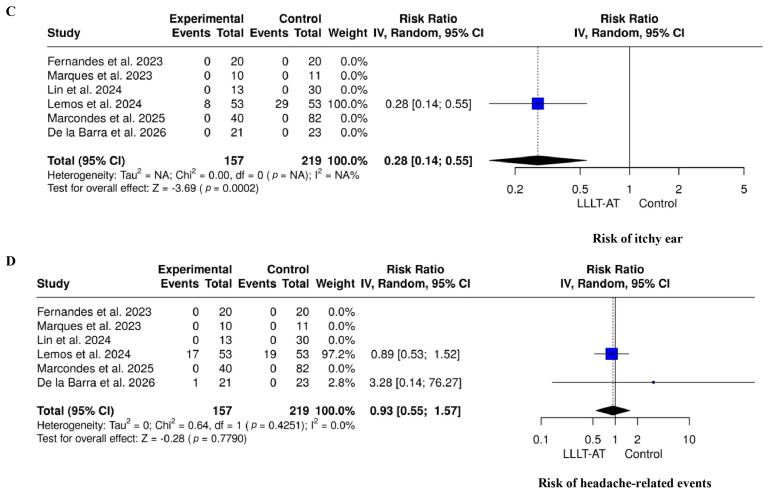
Forest plot for adverse eventsfollowing LLLT-AT interventions. (**A**) Comparison of the risk of ear pain between LLLT-AT and control interventions. (**B**) Comparison of the risk of ear hyperemia between LLLT-AT and control interventions. (**C**) Comparison of the risk of itchy ear between LLLT-AT and control interventions. (**D**) Comparison of the risk of headache-related events between LLLT-AT and control interventions [24,25,46,47,48,49].

*Adverse events.* Figure 7A–D present the analyses of adverse events associated with LLLT-AT compared with control interventions [24,25,46,47,48,49]. Reported adverse events included ear pain, ear hyperemia, itchy ear, and intervention-related headache. Dichotomous outcomes were analyzed using Mantel–Haenszel RRs with 95% confidence intervals. Studies reporting zero events in both groups were retained descriptively and did not contribute to pooled RR estimates, according to Cochrane recommendations [40]. Most adverse-event estimates were based on a limited number of informative comparisons, with a substantial contribution from Lemos et al. [47], and should therefore be interpreted cautiously.

Figure 7A demonstrated a significantly lower risk of ear pain in the LLLT-AT group compared with control interventions (RR = 0.13; 95% CI: 0.06 to 0.28; *p* < 0.01) [47]. Similarly, Figure 7B showed a reduced risk of ear hyperemia associated with LLLT-AT (RR = 0.32; 95% CI: 0.18 to 0.59; *p* < 0.01). Figure 7C also demonstrated a lower risk of itchy ear in participants receiving LLLT-AT compared with controls (RR = 0.28; 95% CI: 0.14 to 0.55; *p* < 0.01). Figure 7D presents the meta-analysis of headache-related adverse effects evaluated across two studies [46,49]. The pooled analysis demonstrated no statistically significant difference between LLLT-AT and control interventions (RR = 0.93; 95% CI: 0.55 to 1.57; *p* = 0.7790).

According to the GRADE assessment, the certainty of evidence was low for all adverse event outcomes, including ear pain, ear hyperemia, itchy ear, and headache-related events (Table 3) [36].

### 3.7. Publication Bias

Publication bias was not assessed because fewer than 10 studies were available for the primary outcome, which precluded reliable evaluation using funnel plots and Egger’s regression test, in accordance with current methodological recommendations [40,51].

## 4. Discussion

This systematic review synthesized the current evidence regarding the effects of LLLT-AT on anxiety and related outcomes across heterogeneous clinical populations. Overall, pooled analyses demonstrated reductions in post-treatment anxiety symptoms favoring LLLT-AT compared with control interventions; however, sensitivity analyses excluding studies with high RoB resulted in an attenuated and non-significant effect estimate, indicating limited robustness of the primary finding. In addition, the substantial heterogeneity and very low-to-low certainty of evidence warrant cautious interpretation. Furthermore, the loss of statistical significance after excluding studies with high risk of bias indicates that the observed anxiolytic effect is sensitive to methodological quality and should therefore be considered preliminary rather than confirmatory. In studies involving temporomandibular disorders, favorable effects were also observed for pain intensity and disability outcomes, whereas adverse eventssuch as ear pain, hyperemia, and itchy ear were less frequent among participants receiving LLLT-AT.

### 4.1. Neurophysiological Mechanisms of LLLT-AT

The potential anxiolytic effects of LLLT-AT remain insufficiently established at the mechanistic level. Current explanations are largely based on indirect evidence from auricular stimulation, vagal modulation, needle-based auriculotherapy, and broader photobiomodulation research rather than on direct mechanistic studies of LLLT-AT. Therefore, the mechanisms discussed below should be interpreted as plausible hypotheses rather than confirmed pathways. Importantly, direct evidence involving LLLT-AT is currently limited mainly to clinical outcomes, treatment acceptability, safety, and intervention parameters reported in randomized controlled trials [24,25,46,47,48,49], whereas the underlying neurobiological mechanisms remain largely inferential and require direct experimental confirmation. From a theoretical perspective, these potential effects may be partially explained by neurophysiological mechanisms associated with both auriculotherapy and photobiomodulation. Auricular interventions involve stimulation of specific acupoints located in a highly innervated region connected to autonomic and central nervous system pathways through branches of the vagus, trigeminal, facial, and glossopharyngeal nerves [11,12,52]. Through these neuroanatomical connections, auricular stimulation may influence emotional regulation, stress responses, autonomic balance, and nociceptive processing [53]. In particular, the auricular branch of the vagus nerve has been proposed as a potential pathway underlying the autonomic and emotional modulation associated with auricular stimulation [11,12]. However, recent perspectives on auricular neuromodulation suggest that the physiological effects of auricular stimulation may extend beyond isolated vagal activation, involving interactions between vagal, trigeminal, cervical, and central autonomic pathways [54]. These pathways have been proposed to influence central mechanisms involved in autonomic regulation, affective processing, stress responses, and nociceptive modulation [11,53,55,56]. Such interactions may be relevant in conditions characterized by overlapping emotional and somatic components, although their specific role in LLLT-AT remains to be established [24,25,46,48,49].

Previous evidence has suggested that auricular stimulation may influence autonomic nervous system activity, stress responses, and limbic-related pathways involved in anxiety processing [11,53]. Functional neuroimaging studies have reported changes in brain activity following auricular stimulation, supporting potential interactions with central nervous system structures associated with emotional and autonomic regulation [55,56]. Moreover, vagal activation induced by auricular stimulation has been associated with modulation of the cholinergic anti-inflammatory pathway, oxidative stress responses, and autonomic balance, mechanisms that may collectively contribute to reductions in stress-related physiological responses and anxiety-related symptoms [57].

In parallel, laser photobiomodulation may provide an additional biological pathway through which LLLT-AT could influence anxiety- and stress-related responses. LLLT irradiation has been associated with mitochondrial activation, cytochrome c oxidase activity, ATP synthesis, modulation of oxidative stress, and regulation of inflammatory mediators [18,58,59]. When applied to auricular acupoints, these effects may theoretically interact with autonomic and central regulatory pathways involved in stress modulation. However, these mechanisms remain incompletely understood, and their specific contribution to anxiety-related outcomes following LLLT-AT requires further investigation [18,57].

Across the included studies, relative consistency was observed in the selection of auricular acupoints traditionally associated with emotional regulation and autonomic balance, particularly Shenmen, Sympathetic Nervous System, Kidney, Heart, Brainstem, and Liver-related points [17,24,25,46,47,48,49]. These acupoints have been commonly incorporated into auriculotherapy protocols targeting anxiety, stress-related symptoms, sleep disturbances, and pain-related conditions [11,12,53,57]. Furthermore, recent evidence involving auricular laser acupuncture has reported improvements in anxiety, stress, fatigue, and sleep-related outcomes while maintaining high treatment acceptability and minimal adverse reactions [47]. However, the specific contribution of individual acupoints and the neurophysiological pathways underlying LLLT-AT effects remain uncertain and require further investigation.

Beyond anxiety-related modulation, the interaction between auricular neuromodulation and photobiomodulation may also contribute to pain modulation through autonomic regulation, inflammatory mechanisms, and central nociceptive processing pathways [12,17,18,58]. These mechanisms may partially explain the favorable findings observed for pain intensity and disability outcomes in temporomandibular disorder populations included in this review; however, their specific contribution within LLLT-AT interventions remains to be established [12,18,58,59].

### 4.2. Comparator-Related Considerations

Interestingly, subgroup analyses demonstrated greater effects when LLLT-AT was compared with placebo or no-treatment controls, whereas comparisons with active interventions showed less consistent findings [24,25,46,47,48,49]. These results should be interpreted cautiously because several active comparators included interventions with potential therapeutic effects on anxiety-related symptoms, such as needle-based auriculotherapy and multidisciplinary supportive care in women undergoing chemotherapy for advanced breast cancer [47,48]. In these clinical contexts, emotional support, symptom management, and therapeutic interaction may also contribute to improvements in anxiety-related outcomes [10,53]. Nevertheless, the favorable safety and tolerability profile observed for LLLT-AT may represent a clinically relevant advantage over more invasive auriculotherapy approaches, particularly in individuals with heightened pain sensitivity, fear of needles, or poor tolerance to invasive procedures [14]. Future high-quality RCTs using rigorous sham-controlled and active-comparator designs are needed to better clarify the specific effects and potential clinical advantages of LLLT-AT interventions.

### 4.3. Clinical Heterogeneity and Protocol Variability

Although anxiety symptoms constituted the common outcome across studies, the included populations differed substantially in clinical context, psychosocial burden, comorbidity profile, and intervention setting. Therefore, the pooled anxiety estimate should be interpreted as an exploratory synthesis across heterogeneous anxiety-related contexts rather than as evidence for a uniform effect of LLLT-AT across all populations. The investigated populations represented markedly different clinical contexts, including individuals with temporomandibular disorders and anxiety, parental perioperative anxiety, university students, and women with advanced breast cancer undergoing chemotherapy [24,25,46,47,48,49]. These populations likely differed in baseline emotional distress, psychosocial burden, stress-related physiological responses, comorbidities, and responsiveness to non-pharmacological interventions. Such variability may have contributed to the considerable heterogeneity observed across pooled anxiety analyses and supports the indirectness concerns identified in the GRADE assessment.

Laser dosimetry and treatment protocols varied considerably across the included studies. Wavelengths ranged from 660 to 905 nm, with both continuous and pulsed emission modes employed. These differences may be biologically relevant because wavelength influences light penetration and photon absorption by different chromophores within auricular tissues, potentially resulting in distinct photobiomodulatory responses [58,59]. Likewise, total delivered energy ranged from 9 to 64 J, together with substantial variation in energy density, treatment duration, and the number of irradiated auricular points. Such dosimetric variability may have influenced treatment effects and contributed to the observed between-study heterogeneity [59]. Future head-to-head comparative RCTs are warranted to determine whether specific wavelengths and dosimetric parameters are associated with superior clinical outcomes and to support the development of standardized treatment protocols.

In addition, multiple anxiety assessment instruments were used across studies, including GAD-7, STAI-S, BAI, and NRS-Anxiety, which may have further increased variability in pooled effect estimates. Comparator interventions also varied substantially across the included studies, including no treatment, placebo LLLT-AT, stimulation of non-anxiolytic auricular points, active needle auriculotherapy, and placebo LLLT-AT combined with concomitant therapies such as chemotherapy, multidisciplinary care, or myofascial release [24,25,46,47,48,49]. This methodological variability, together with the marked heterogeneity of the included clinical populations and the use of concomitant interventions, may have influenced treatment responses and introduced potential confounding, limiting attribution of the observed effects exclusively to LLLT-AT and contributing to the observed between-study heterogeneity. Similar methodological variability has previously been reported in the auriculotherapy literature addressing anxiety, stress, and depression outcomes [17,53,57]. Consequently, although pooled analyses demonstrated favorable post-treatment findings, heterogeneity among clinical populations, intervention protocols, and outcome assessment methods limits the direct generalizability and certainty of the current evidence. At this stage, LLLT-AT should be considered a candidate complementary intervention to be further evaluated for anxiety-related symptoms, particularly in individuals presenting concomitant pain conditions, emotional distress, or poor tolerance to needle-based interventions. However, the methodological heterogeneity of the included studies currently limits reproducibility and precludes identification of an optimal therapeutic protocol. Given the limited number of trials, substantial heterogeneity, and very low-to-low certainty of evidence for anxiety outcomes, the clinical and public health implications of these findings should be considered hypothesis-generating and should not be interpreted as direct clinical or public health recommendations. Therefore, future high-quality RCTs should prioritize standardized intervention protocols, rigorous dosimetric reporting, and longer-term follow-up assessments to improve reproducibility, strengthen the certainty of the evidence, and inform future clinical research and implementation strategies.

### 4.4. Dosimetric and Protocol Considerations

Current evidence remains insufficient to establish definitive dosimetric recommendations for LLLT-AT in anxiety-related conditions. Nevertheless, the protocols identified in this systematic review provide a preliminary overview of the dosimetric parameters currently investigated. Across the included studies, wavelengths ranged from 660 to 905 nm, energy density from 0.19 to 4 J/cm^2^, the number of irradiated auricular points from 4 to 16, and irradiation time from 20 to 56 s per point [24,25,46,47,48,49]. These ranges should be interpreted as a descriptive summary of the dosimetric approaches reported in the existing literature, rather than as evidence-based recommendations for optimal LLLT-AT dosing. Future randomized controlled trials should determine whether specific dosimetric parameters are associated with superior clinical outcomes while prioritizing standardized photobiomodulation reporting, dose–response analyses, protocol harmonization, and longer-term follow-up assessments.

### 4.5. Adverse Eventsand Safety Profile

An additional relevant finding of this review was the favorable safety profile observed for LLLT-AT. Pooled analyses demonstrated significantly lower risks of ear pain, hyperemia, and itchy ear compared with control interventions, suggesting that LLLT-AT may represent a well-tolerated auriculotherapy approach [24,25,46,47,48,49]. The non-invasive and painless nature of LLLT-AT may partially explain these findings, as laser auriculotherapy does not require skin penetration and may reduce local tissue irritation, discomfort, and procedure-related apprehension compared with needle-based auricular interventions [14,17]. Notably, studies comparing LLLT-AT with acupuncture reported a greater frequency of local adverse reactions in needle-based groups [21,47].

These findings may be clinically relevant in populations presenting heightened pain sensitivity, emotional distress, procedural anxiety, or cancer-related symptom burden, supporting the potential role of LLLT-AT as a non-invasive supportive intervention within integrative care settings [60]. Previous qualitative evidence has suggested that treatment comfort, perceived safety, and reduced fear of needles may facilitate patient acceptance and adherence to auriculotherapy interventions [11,14,61]. Furthermore, auriculotherapy interventions are frequently described as relaxing and calming experiences that may support emotional well-being and treatment adherence [14,60]. These findings are also consistent with emerging evidence suggesting that laser-based auricular interventions may provide clinically relevant improvements while maintaining favorable tolerability and non-invasive application characteristics compared with needle-based approaches [21,47]. However, adverse event reporting remained inconsistent across studies, highlighting the need for standardized safety monitoring in future RCTs. In addition, although no ocular adverse events were reported, appropriate laser safety measures, including avoidance of direct ocular exposure and use of eye protection when required, should be considered due to the anatomical proximity of auricular points to the eyes.

### 4.6. Limitations

Several limitations should be considered when interpreting the findings of this review. The number of available RCTs remained limited, and substantial clinical and methodological heterogeneity was identified across studies, including differences in clinical populations, anxiety assessment instruments, laser dosimetry, intervention protocols, comparator interventions, and follow-up periods. In addition, some studies presented methodological concerns related to RoB [34]. Sensitivity analysis excluding studies with high RoB resulted in an attenuated and non-significant effect estimate for anxiety outcomes, indicating that the magnitude of the observed anxiolytic effect should be interpreted cautiously. An additional consideration is that the first and corresponding author of this systematic review was also the lead author of one of the included RCTs [49]. This authorial overlap was addressed by applying the same prespecified eligibility, data extraction, PEDro methodological quality assessment, and RoB assessment procedures used for all included studies, with data extraction, PEDro scoring, and RoB judgments reviewed by the other reviewers and resolved by consensus. Moreover, adverse event reporting and longer-term follow-up assessments were inconsistently reported across trials. Publication bias could not be formally assessed because fewer than 10 studies were available for quantitative synthesis [51]. Consequently, the certainty of evidence ranged from very low to low across outcomes.

Nevertheless, this systematic review was conducted in accordance with PRISMA 2020 recommendations and included a comprehensive search strategy across eight major electronic databases [26,29], strengthening the methodological rigor of the evidence synthesis. Furthermore, this study represents the first systematic review and meta-analysis specifically evaluating LLLT-AT for anxiety-related symptoms, providing a preliminary framework for future research and protocol development in this emerging field.

### 4.7. Public Health Implications

Anxiety disorders represent a major public health concern because of their high prevalence, chronic course, disability burden, healthcare costs, and frequent under recognition and undertreatment [2]. In this context, evidence-informed complementary strategies may be relevant when they are safe, acceptable, feasible, and integrated within established models of care [10]. Auriculotherapy has shown favorable implementation characteristics in primary health care, including scalability of professional training, high user acceptance, and perceived clinical benefits [14,60,62]. These characteristics support further investigation of LLLT-AT as a potential complementary strategy within multidisciplinary healthcare contexts, particularly when safe, non-invasive, and acceptable therapeutic options are being explored [17,62]. However, considering the limited number of available trials, substantial heterogeneity, and very low-to-low certainty of evidence, the potential public health implications of LLLT-AT remain preliminary and should not be interpreted as recommendations for implementation into routine healthcare settings. Further effectiveness and implementation research is required before its role in healthcare systems can be established.

## 5. Conclusions

The current evidence suggests that LLLT-AT may be associated with reductions in anxiety symptoms across different clinical populations; however, these findings should be considered as preliminary due to the limited number of available RCTs, substantial clinical and methodological heterogeneity, and low certainty of evidence for anxiety outcomes. Importantly, sensitivity analyses excluding studies with high risk of bias showed that the pooled effect for anxiety was not statistically significant, indicating that the robustness of the current evidence remains limited. Favorable effects were also observed for pain intensity and disability outcomes. In addition, the available evidence suggests that LLLT-AT may represent a safe, well-tolerated, and non-invasive alternative to other auriculotherapy approaches, demonstrating lower risks of selected local adverse eventscompared with control interventions. However, the substantial heterogeneity in clinical populations, intervention protocols, and dosimetric parameters and very low-to-low certainty of evidence require cautious interpretation of the current findings. Future high-quality RCTs are warranted to confirm these preliminary findings and reduce the remaining uncertainty regarding the effectiveness of LLLT-AT for anxiety-related symptoms.

## Figures and Tables

**Table 1:** Characteristics of included studies.

First Author (Year) Country	PEDro Score	Selection Criteria	Participants (*n*) Mean Age (SD)	Groups (*n*)	Sessions	Outcomes (Instrument)	Assessment Instances	Results After Treatment	Study Conclusion	Adverse Events	Source of Funding
Fernandes et al. (2023) [24] Brazil	5/10	Inclusion Criteria: - Patients with one or more TMD symptoms - Age between 20–45 years Exclusion Criteria - Edentulous patients -Total prosthesis users - Smokers - Alcohol consumption - Illicit drug use - Continuous use of medications for sleep or anxiety alterations	40 (33 F, 7 M) 29.9 (7.0) years Dropout 0	EG (20): LLLT-AT CG (20): No treatment	1 s/week for 10 weeks	(A) AL (BAI) (B) SQ interference (Fletcher and Luckett questionnaire) (C) Degree of TMD (RDC/TMD questionnaire)	T0: baseline T1: post-treatment (10 weeks)	EG: ↓ AL * and ↓ SQ interference CG: ↓ AL and ↓ SQ interference EG < CG for AL * CG < EG for SQ interference	LLLT-AT reduced anxiety but showed no effect on sleep disorders or TMD symptoms	None	Unified Scholarship Program for the Support and Training of Graduation Students—University of São Paulo (PUB-USP)
Marques et al. (2023) [25] Brazil	7/10	Inclusion criteria: - Diagnosis of TMD (DC/TMD protocol) Exclusion criteria: - Ear piercing or birthmark - History of neoplasia - Pregnancy or lactation - Cardiac arrhythmia	21 (NS F, NS M) 39.0 (16.4) years Dropout 1	EG (10): LLLT-AT CG (11): LLLT-AT placebo	1 s/week for 4 weeks	(A) PI (GCPS) (B) Disability (JFLS-8) (D) Mandibular movements (MMM) (E) AL (GAD-7) (D) Anxiety and depression (PHQ-4)	T0: baseline T1: post-treatment (week 4)	EG: ↓ PI *, ↓ disability *, ↓ anxiety *, ↑ mandibular movements, and ↓ health status alteration * CG: ↓ anxiety *, and no change in PI, disability, mandibular movements and health status EG < CG for PI *, ↓ disability*, ↓ anxiety *, ↑ mandibular movements, and ↓ health status alteration *	LLLT-AT demonstrated feasibility with positive effects on TMD-related pain and disability, while outcomes on anxiety and general health remain inconclusive.	None	Coordination for the Improvement of Higher Education Personnel—Brazil (CAPES)—Funding Code 001, and Federal University of Alfenas—UNIFAL-MG.
Lin et al. (2024) [46] USA	6/10	Inclusion criteria: - English-speaking parents - No major medical or psychiatric illness - No prior acupuncture experience Exclusion criteria: - Child undergoing emergency surgery - Parent unable to read/speak English	43 (NS F, NS M) 40.3 (16.4) years Dropout 2	EG (13): LLLT-AT (anxiolytic acupoints) CG1 (15): LLLT-AT (non-anxiolytic acupoints) CG2 (15): LLLT-AT placebo	1 s/week for 1 week	(A) AL (STAI)—only for baseline assessment (B) AL (NRS-anxiety) (C) Perceived treatment acceptability (UAFQ)	T0: baseline T1: post-treatment T2: follow-up (30 min post-treatment)	EG: ↓ AL * CG1: ↓ AL CG2: ↓ AL * EG < CG2 < CG1 for AL *	LLLT-AT reduced parental anxiety after 30 min, with no adverseevents.	None	Susan Samueli Integrative Health Institute, Irvine, California, USA
Lemos et al. (2024) [47] Brazil	7/10	Inclusion criteria: - Students ≥18 years - Availability for assessments and treatments Exclusion criteria: - Refusal of random allocation - Rejection/fear of interventions -Allergy to micropore tape or metal - History of photosensitivity or skin cancer (head/neck) - Compromised immune system or epilepsy - Chemical peeling or isotretinoin use within 6 months - Ear piercings near selected points - Use of hearing aids or cardiac pacemaker - Ear tattoos, injuries, inflammation, or deformities - Pregnancy, lactation, or planning pregnancy - Receipt of any PICS within the last 3 months	115 (88 F, 26 M) 22.6 (4.6) years Dropout 8	EG (58): LLLT-AT CG (57): AT with needles	1 s/week for 5 weeks	(A) AL (STAI) (B) Heart rate (portable digital oximeter) (C) Respiratory rate (chest and/or abdomen observation) (D) Treatment satisfaction (Likert scale)	T0: baseline T1: post-treatment (week 5) T2: follow-up (week 6)	EG: ↓ AL *, ↓ heart rate, and ↓ respiratory rate CG: ↓ AL *, ↓ heart rate *, and ↓ respiratory rate * EG = CG for AL, heart and respiratory rate	Both needle and LLLT-AT reduced anxiety. No group differences in physiological parameters. Laser caused fewer adverse events than needles	Needle acupuncture caused more minor adverse events (pain, hyperemia, itching, and headache) than LLLT-AT	Not reported
Marcondes et al. (2025) [48] Brazil	8/10	Inclusion criteria: - Women aged ≥18 years - Diagnosis of advanced breast cancer - Receiving first- or second-line palliative chemotherapy Exclusion criteria: - Hospitalized patients - Use of other NPTs	123 (123 F, 0 M) 53.3 (11.3) years Dropout 3 (disease)	EG (41): LLLT-AT + chemotherapy + multidisciplinary care CG1 (40): LLLT-AT placebo + chemotherapy + multidisciplinary care CG2 (42): Chemotherapy + multidisciplinary care	1 s/week for 5 weeks	(A) AL (STAI) (B) HRQoL (QLQ-C30) (C) Functionality (FACT) (D) Fatigue (FACIT-F)	T0: baseline T1: during treatment (week 5) T2: post-treatment (week 10)	EG: ↓ AL *, ↑HRQoL *, ↓ Fatigue * and ↑Functionality * CG1: ↓ AL *, ↑HRQoL *, ↓ Fatigue * and ↑Functionality * CG2: ↓ AL *, ↑HRQoL *, ↓ Fatigue and ↑Functionality * EG = CG1 = CG2 for AL EG < CG1 < CG2 for Interference in HRQoL * and ↓ Fatigue * EG = CG1 = CG2 for Functionality	Improved HRQoL and fatigue during and after treatment (from week 5). No effect on anxiety; safe, feasible, low-cost, with no adverse events.	None	No funding
De la Barra et al. (2026) [49] Chile	9/10	Inclusion criteria - Adults ≥18 years - Both sexes - Myogenic TMD diagnosed according to DC/TMD - Masticatory myalgia/myofascial pain - ≥1 pain episode during the previous 30 days - GAD-7 score ≥ 5 Exclusion criteria - Recent cervical musculoskeletal injury - Auricular skin lesions/diseases - Anti-inflammatory or photosensitizing drug use - Auricular tattoos - Cancer/tumor history (<5 years) - Fitzpatrick phototype V–VI - Autoimmune disease, porphyria, or pellagra - Epilepsy - Current occlusal splint use	44 (25 F, 19 M) 23.8 (5.5) years Dropout: 3 participants (withdrawal/headache)	EG (21): LLLT-AT + myofascial release CG (23): LLLT-AT placebo + myofascial release	2 s/week for 3 weeks	(A) PPT (pressure algometry) (B) Anxiety (GAD-7) (C) MMOROM (metric ruler) (D) Mandibular functional limitation (JFLS-8)	T0: baseline T1: post-treatment (3 weeks) T2: follow-up (4 weeks after intervention)	EG: ↑PPT *, ↓ Anxiety *, ↑MMOROM *, and ↓ JFLS-8 * CG: ↑PPT *, ↓ Anxiety *, ↑MMOROM *, and ↓ JFLS-8 * EG > CG for Anxiety * EG = CG for PPT, MMOROM, and JFLS-8	LLLT-AT combined with myofascial release reduced anxiety symptoms but did not provide additional benefits for PPT, MMOROM, or mandibular function compared with sham LLLT-AT combined with myofascial release.	Mild headache reported in 1 participant (dropout)	Vice-Rectorate for Research, Universidad Andrés Bello, Chile (Grant No. DI-03-25/CBC)

**Abbreviations:** AL—Anxiety Level; AT—Auriculotherapy; BAI—Beck Anxiety Inventory; CG—Control Group; EG—Experimental Group; FACT—Functional Assessment of Cancer Therapy; FACIT-F—Functional Assessment of Chronic Illness Therapy: Fatigue Questionnaire; GAD-7—Generalized Anxiety Disorder-7; GCPS—Graded Chronic Pain Scale; HRQoL—Health-Related Quality of Life; JFLS-8—Jaw Functional Limitation Scale-8; LLLT-AT—Low-Level Laser Auriculotherapy; MMM—Mandibular Movement Measurements; MMOROM—Maximum Mouth Opening Range of Motion; NRS—Numeric Rating Scale; PHQ-4—Patient Health Questionnaire-4; PI—Pain Intensity; PICS—Integrative and Complementary Health Practices; PPT—Pressure Pain Threshold; QLQ-C30—Quality of Life Questionnaire Core-30; RDC/TMD—Research Diagnostic Criteria for Temporomandibular Disorders; SQ—Sleep Quality; STAI—State–Trait Anxiety Inventory; TMD—Temporomandibular Disorders; UAFQ—Usability and Acceptability Feedback Questionnaire. *p* < 0.05 *. ↑ denotes an increase and ↓ denotes a decrease in the corresponding outcome.

**Table 2:** Laser parameters reported in the included studies.

Characteristics/Parameters	Fernandes et al. (2023) [24]	Marques et al. (2023) [25]	Lin et al. (2024) [46]	Lemos et al. (2024) [47]	Marcondes et al. (2025) [48]	De la Barra et al. (2026) [49]
Laser model	Therapy EC Duo Mom^®^	Ecco Fibra Laser^®^	NS	Ilib Laser^®^	ACP Therapy DCM^®^	COMBI 400L (As-Ga-Al diode)
Wavelength (nm)	808 nm	660 nm	780 nm	660 nm ± 10 nm	880 nm	905 nm
Emission mode (continuous/pulsed)	NS	Pulsed (pulse frequency 9.12 Hz)	NS	Continuous	Continuous	Pulsed
Peak power (W)	0.1 W	NS	NS	NS	NS	13.5 W
Mean power (mW)	100 mW	100 mW		100 mW ± 20%	100 mW	71 mW
Spot size (cm^2^)	NS	1 cm^2^	0.1 cm^2^	NS	1 cm^2^	0.8 cm^2^
Number of acupoints	8	16	6	11	8	4
Treatment protocol	Punctual application at 8 points: - Upper limb - Maxilla - Anxiety - Shenmen - Point zero - Stomach - Mandible - Stress	Punctual application on 16 points: - Point Zero (O′) - Emotional scar point: 10 points - Cervical rib: 3 points - ACTH - TMJ	Punctual application on 6 points: - Relaxation - Shenmen - Point Zero (O′) - Extraneous point - Wrist - Shoulder	Punctual application on 11 points: - Shenmen - Kidney - Autonomic Nervous System - Brain stem - Spleen - Anterior anxiety - Diaphragm - Muscle relaxation - Sleep - Lung - Adrenal points	Punctual application at 8 points: - Shenmen - Kidney - Autonomic Nervous System - Liver - Spleen - Brain stem - Lung 1 - Heart - Liver 2	Punctual application at 4 auricular points: - Shenmen - Kidney - Liver - Point Zero (O′)
Energy density (J/cm^2^)	NS	4 J/cm^2^	0.19 J/cm^2^	NS	4 J/cm^2^	4 J/cm^2^
Total energy (J)	NS	64 J (4 J per point)	9 J (1.5 J per point)	22 J (2 J per point)	32 J (4 J per point)	16 J
Treatment time (s)	NS	40 s per point (total 640 s)	50 s per point (total 300 s)	20 s per point (total 220 s)	40 s per point (total 320 s)	56 s per point (224 s total)

**Abbreviations:** ACTH—adrenocorticotropic hormone; cm—centimeters; cm^2^—centimeters squared; J—Joules; nm—nanometers; NS—not specified; sec—seconds; TMJ—temporomandibular joint.

**Table 3:** Summary of findings and certainty of evidence (GRADE) for anxiety and related outcomes.

Certainty Assessment							№ of Patients		Effect Estimate (95% CI)	Certainty ^f^	Importance ^g^
Studies	Study Design	Risk of Bias	Inconsistency	Indirectness	Imprecision	Publication Bias	LLLT-AT Group	Control Groups			
**Anxiety outcome (BAI): LLL-AT vs. No treatment**											
1 [24]	RCTs	serious ^a^ (−1)	not serious ^b^ (0)	serious ^c^ (−1)	serious ^d^ (−1)	Not assessed ^e^	**20**	**20**	**SMD = −0.94**	⨁⨁◯◯ Low	CRITICAL
									(−1.60 to −0.29)		
**Anxiety outcome (GAD-7, NRS-Anxiety, and STAI-S): LLLT-AT vs. Placebo**											
4 [25,46,47,48,49]	RCTs	serious ^a^ (−1)	serious ^b^ (−1)	serious ^c^ (−1)	serious ^d^ (−1)	Not assessed ^e^	58	89	**SMD = −0.76**	⨁◯◯◯ Very Low	CRITICAL
									(−1.51 to −0.00)		
**Anxiety outcome (GAD-7, NRS-Anxiety, and STAI-S): LLLT-AT vs. Active control**											
3 [46,47,48]	RCTs	serious ^a^ (−1)	serious ^b^ (−1)	serious ^c^ (−1)	serious ^d^ (−1)	Not assessed ^e^	80	111	**SMD = −0.49**	⨁◯◯◯ Very Low	CRITICAL
									(−1.61 to 0.63)		
**Pain intensity (GCPS and pain domain of the QLQ-C30): LLLT-AT vs. Placebo**											
2 [25,48]	RCTs	serious ^a^ (−1)	not serious ^b^ (0)	serious ^c^ (−1)	serious ^d^ (−1)	Not assessed ^e^	30	51	**SMD = −0.82**	⨁⨁◯◯ Low	CRITICAL
									(−1.29 to −0.34)		
**Pain intensity (pain domain of the QLQ-C30): LLLT-AT vs. Placebo**											
1 [48]	RCTs	serious ^a^ (−1)	not serious ^b^ (0)	serious ^c^ (−1)	serious ^d^ (−1)	Not assessed ^e^	21	43	**SMD = −0.82**	⨁⨁◯◯ Low	CRITICAL
									(−1.36 to −0.28)		
**Disability (JFLS-8): LLLT-AT vs. Placebo**											
2 [25,49]	RCTs	serious ^a^ (−1)	not serious ^b^ (0)	serious ^c^ (−1)	serious ^d^ (−1)	Not assessed ^e^	31	26	**SMD = −0.64**	⨁⨁◯◯ Low	CRITICAL
									(−1.19 to −0.46)		
**Anxiety outcome (GAD-7 and BAI): LLLT-AT versus placebo or no treatment in individuals with TMD-related anxiety**											
3 [24,25,49]	RCTs	serious ^a^ (−1)	not serious ^b^ (0)	serious ^c^ (−1)	serious ^d^ (−1)	Not assessed ^e^	51	54	**SMD = −1.01**	⨁⨁◯◯ Low	CRITICAL
									(−1.51 to −0.68)		
**Anxiety outcome (NRS-Anxiety): LLLT-AT versus sham or placebo in parental perioperative anxiety**											
1 [46]	RCTs	serious ^a^ (−1)	not serious ^b^ (0)	serious ^c^ (−1)	serious ^d^ (−1)	Not assessed ^e^	13	30	**SMD = −1.45**	⨁⨁◯◯ Low	CRITICAL
									(−2.18 to −0.71)		
**Anxiety outcome (STAI-S): LLLT-AT versus control in university student anxiety**											
1 [47]	RCTs	serious ^a^ (−1)	not serious ^b^ (0)	serious ^c^ (−1)	serious ^d^ (−1)	Not assessed ^e^	53	53	**SMD = 0.07**	⨁⨁◯◯ Low	CRITICAL
									(−0.31 to 0.45)		
**Anxiety outcome (STAI-S): LLLT-AT versus control (placebo, chemotherapy and multidisciplinary care) in breast cancer-related anxiety**											
1 [48]	RCTs	serious ^a^ (−1)	not serious ^b^ (0)	serious ^c^ (−1)	serious ^d^ (−1)	Not assessed ^e^	41	83	**SMD = 0.10**	⨁⨁◯◯ Low	CRITICAL
									(−0.28 to 0.47)		
**Ear pain: LLLT-AT vs. Controls**											
6 [24,25,46,47,48,49]	RCTs	serious ^a^ (−1)	not serious ^b^ (0)	serious ^c^ (−1)	serious ^d^ (−1)	Not assessed ^e^	157	219	**RR = 0.13**	⨁⨁◯◯ Low	IMPORTANT
									(0.06 to 0.28)		
**Ear hyperemia: LLLT-AT vs. Controls**											
6 [24,25,46,47,48,49]	RCTs	serious ^a^ (−1)	not serious ^b^ (0)	serious ^c^ (−1)	serious ^d^ (−1)	Not assessed ^e^	157	219	**RR = 0.32**	⨁⨁◯◯ Low	IMPORTANT
									(0.18 to 0.59)		
**Itchy ear: LLLT-AT vs. Controls**											
6 [24,25,46,47,48,49]	RCTs	serious ^a^ (−1)	not serious ^b^ (0)	serious ^c^ (−1)	serious ^d^ (−1)	Not assessed ^e^	157	219	**RR = 0.28**	⨁⨁◯◯ Low	IMPORTANT
									(0.14 to 0.55)		
**Headache-related events: LLLT-AT vs. Controls**											
6 [24,25,46,47,48,49]	RCTs	serious ^a^ (−1)	not serious ^b^ (0)	serious ^c^ (−1)	serious ^d^ (−1)	Not assessed ^e^	157	219	**RR = 0.95**	⨁⨁◯◯ Low	IMPORTANT
									(0.56 to 1.61)		

**Abbreviations:** CI—confidence interval; RCTs—randomized controlled trials; RR—risk ratio; SMD—standardized mean difference. **Explanations:** ^a^ Overall risk of bias was influenced by concerns regarding outcome measurement and selective reporting, with 33% of studies rated as high risk of bias; ^b^ Evidence certainty was downgraded for inconsistency in the presence of substantial heterogeneity (I^2^ > 50%); ^c^ Evidence certainty was downgraded for indirectness because the included studies enrolled heterogeneous clinical populations and anxiety-related contexts, limiting the direct applicability of the findings to a single target population; ^d^ Imprecision was evaluated based on the width of the confidence intervals (CIs), crossing of the line of no effect, and total sample size across studies (<400 participants); ^e^ Publication bias was not assessed because fewer than 10 studies were included; ^f^ Certainty of evidence (GRADE): ⨁⨁◯◯ = low certainty (the true effect may be substantially different from the estimated effect); ⨁◯◯◯ = very low certainty (the true effect is likely to be substantially different from the estimated effect). ^g^ Outcome importance was defined a priori according to GRADE guidance and clinical relevance for decision-making.

## Data Availability

The original contributions presented in this study are included in the article and Supplementary Materials. Data are available from the corresponding author upon reasonable request.

## Supplementary Materials

The following supporting information can be downloaded at: https://www.mdpi.com/article/10.3390/ijerph23070919/s1, Supplementary Table S1. Search strategy; Supplementary Table S2. Summary of excluded articles via databases, registries, and other methods; Supplementary Table S3. Methodological quality of included studies assessed using the PEDro scale; Supplementary File S1: PRISMA 2020 Checklist. Refs. [24,25,26,46,47,48,49] are cited in the Supplementary Materials.

## Abbreviations

The following abbreviations are used in this manuscript:

AT	Auriculotherapy
BAI	Beck Anxiety Inventory
CI	Confidence Interval
CINAHL	Cumulative Index to Nursing and Allied Health Literature
D1–D5	Domains of the RoB 2 tool
EMHS	Eastern Metropolitan Health Service
GAD-7	Generalized Anxiety Disorder-7
GCPS	Graded Chronic Pain Scale
GRADE	Grading of Recommendations Assessment, Development and Evaluation
H	Heterogeneity statistic H
HRQoL	Health-Related Quality of Life
I^2^	Higgins inconsistency statistic
IQR	Interquartile Range
JFLS-8	Jaw Functional Limitation Scale-8
LLLT	Low-Level Laser Therapy
LLLT-AT	Low-Level Laser Therapy Auriculotherapy
MCIDs	Minimal Clinically Important Differences
MD	Mean Difference
MeSH	Medical Subject Headings
N	Sample Size
NRS	Numeric Rating Scale
NRS-Anxiety	Numeric Rating Scale adapted for anxiety
PBMT	Photobiomodulation Therapy
PEDro	Physiotherapy Evidence Database
PHQ-4	Patient Health Questionnaire-4
PICOS	Population, Intervention, Comparator, Outcomes, and Study Design
PPT	Pressure Pain Threshold
PRISMA	Preferred Reporting Items for Systematic Reviews and Meta-Analyses
PROSPERO	International Prospective Register of Systematic Reviews
Q	Cochran’s Q statistic
Q1–Q3	First and Third Quartiles
QLQ-C30	European Organisation for Research and Treatment of Cancer Quality of Life Questionnaire-Core 30
RCT	Randomized Controlled Trial
REML	Restricted Maximum Likelihood
RoB	Risk of Bias
RoB 2	Risk of Bias 2 tool
RR	Risk Ratio
SD	Standard Deviation
SE	Standard Error
SMD	Standardized Mean Difference
SQ	Sleep Quality
STAI	State–Trait Anxiety Inventory
STAI-S	State Anxiety subscale of the State–Trait Anxiety Inventory
TMJ	Temporomandibular Joint
VAS	Visual Analog Scale
τ	Square root of tau-squared
τ^2^	Between-study variance
