# Supplementary material for: Laser Auriculotherapy for Anxiety Symptoms Across Diverse Populations: A Systematic Review and Meta-Analysis of Randomized Controlled Trials
Source: Int J Environ Res Public Health. 2026 Jul 17;23(7):919. doi: 10.3390/ijerph23070919 (PMC13410117; doi:10.3390/ijerph23070919)
Supplement: Supplementary file 1 [file ijerph-23-00919-s001.zip › Supplementary Tables.pdf]

Supplementary Table S1. Search strategy (last updated July 2, 2026).

Search		Identification of studies via databases and registers									Identification of studies via other methods
	Keywords	PubMed	Scopus	Web of Science	CINAHL	Medline	Science Direct	Cochrane Library	PEDro database	**TOTAL**	Google Scholar
s1	*"Laser Therapy"*	53.198	52.562	16.722	13.245	54.298	24.919	8.533		223477	
s2	*"Low-Level Light Therapy"*	9.405	5.995	909	445	9.405	517	2.062		28738	
s3	*"Photobiomodulation"*	4.863	6.198	6.163	1.646	10.701	3.406	1.919		34896	
s4	*"Photobiomodulation Therapy"*	1.460	1.794	1.758	827	8.453	857	641		15790	
s5	*"Auriculotherapy"*	338	531	353	293	337	570	377		2799	
s6	*"Acupuncture, Ear"*	597	533	124	612	589	215	411		3081	
s7	*S1 OR S2 OR S3 OR S4 OR S5*	61.171	55.801	20.644	15.450	62.274	28.001	11.076		254417	
s8	*"Anxiety"*	401.983	692.886	495.940	155.144	404.050	795.149	98.469		3043621	
s9	*"Anxiety Disorders"*	65.096	168.476	42.688	19.225	66.930	129.610	19.455		511480	
s10	*"Generalized Anxiety Disorder"*	13.314	32.397	19.103	3.916	11.323	41.689	6.114		127856	
s11	*"Anxiety Symptoms"*	20.929	26.169	23.598	9.024	21.026	48.011	6.026		154783	
s12	*S8 OR S9 OR S10 OR S11*	401.983	692.886	495.940	155.144	404.050	48.017	98.469		2296489	
s13	*S6 AND S11*	390	718	298	228	393	2.989	394	0	5410	9.970

*Search algorithm used for formal databases: *("Laser Therapy" OR "Low-Level Light Therapy" OR "Photobiomodulation" OR "Photobiomodulation Therapy" OR "Auriculotherapy" OR "Acupuncture, Ear") AND ("Anxiety" OR "Anxiety Disorders" OR "Generalized Anxiety Disorder" OR "Anxiety Symptoms")* **Search algorithm for other identification of studies via other methods: *("Low-Level Light Therapy" OR "Photobiomodulation" OR "Auriculotherapy" OR "Acupuncture, Ear") AND ("Anxiety" OR "Anxiety Disorders" OR "Generalized Anxiety Disorder")*

Supplementary Table S2. Summary of excluded articles via databases, registries, and other methods.

N°	Source	Author (year)	Reference	Reason of exclusion
1	Identification of studies via other methods	Collet et al. (2008)	Collet J-P, Ducruet T, Robinson AR. Systematic follow-up of a cohort of smokers who received a standard smoking cessation intervention with soft laser therapy. *J Complement Integr Med.* 2008;5(1). http://dx.doi.org/10.2202/1553-3840.1142	LLLT-AT for addiction management (smoking cessation)
2	Identification of studies via other methods	Kerr et al. (2008)	Kerr CM, Lowe PB, Spielholz NI. Low level laser for the stimulation of acupoints for smoking cessation: a double blind, placebo controlled randomised trial and semi structured interviews. *J Chin Med.* 2008;(86):46-51.	LLLT-AT for addiction management (smoking cessation)
3	Identification of studies via other methods	Mostyn et al. (2013)	Mostyn R, Hachey K, Mandel A. Laser acupuncture therapy for the treatment of tobacco addiction. Theralase; 2013 [cited 2026 May 26]. Available from: https://theralase.com/wp-content/uploads/2019/06/Smoking-Cessation-Clinical-Study.pdf	LLLT-AT for addiction management (smoking cessation)
4	Identification of studies via databases and registers	Quah-Smith I. (2014)	Quah-Smith I. Laser ear acupuncture: How much is enough? A prospective observational study on laser dosages required in the healing patient during posturology and during the treatment of mental distress. *Med Acupunct.* 2014;26(3):138–47. http://dx.doi.org/10.1089/acu.2014.1027	Cohort study
5	Identification of studies via other methods	Silva et al. (2014)	Silva R de P, Chaves E de CL, Pillon SC, Silva AM, Moreira D da S, Iunes DH. Contributions of auriculotherapy in smoking cessation: a pilot study. *Rev Esc Enferm USP*. 2014;48(5):883–90. http://dx.doi.org/10.1590/s0080-623420140000500015	LLLT-AT for addiction management (smoking cessation)
6	Identification of studies via databases and registers	Iunes et al. (2015)	Iunes DH, Chaves É de CL, Moura C de C, Côrrea B, Carvalho LC, Silva AM, et al. Role of auriculotherapy in the treatment of temporomandibular disorders with anxiety in university students. *Evid Based Complement Alternat Med.* 2015;2015:430143. http://dx.doi.org/10.1155/2015/430143	Other AT modalities (AT with seeds)
7	Identification of studies via databases and registers	Suen et al. (2016)	Suen LKP, Yeh CH, Yeung SKW. Using auriculotherapy for osteoarthritic knee among elders: a double-blinded randomised feasibility study. *BMC Complement Altern Med.* 2016;16(1):257. http://dx.doi.org/10.1186/s12906-016-1242-6	No interesting outcome (insomnia)

8	Identification of studies via other methods	Sampaio-Filho et al. (2018)	Sampaio-Filho H, Bussadori SK, Gonçalves MLL, da Silva D de FT, Borsatto MC, Tortamano IP, et al. Low-level laser treatment applied at auriculotherapy points to reduce postoperative pain in third molar surgery: A randomized, controlled, single-blinded study. PLoS One. 2018;13(6):e0197989. http://dx.doi.org/10.1371/journal.pone.0197989	LLLT-AT for dental pain
9	Identification of studies via databases and registers	Suen et al. (2019)	Suen LKP, Molassiotis A, Yueng SKW, Yeh CH. Comparison of magnetic auriculotherapy, laser auriculotherapy and their combination for treatment of insomnia in the elderly: A double-blinded randomised trial. *Evid Based Complement Alternat Med*. 2019;2019:3651268. http://dx.doi.org/10.1155/2019/3651268	No interesting outcome (insomnia)
10	Identification of studies via databases and registers	Rodrigues et al. (2019)	Rodrigues M da F, Rodrigues ML, Bueno KS, Aroca JP, Camilotti V, Busato MCA, et al. Effects of low-power laser auriculotherapy on the physical and emotional aspects in patients with temporomandibular disorders: A blind, randomized, controlled clinical trial. *Complement Ther Med*. 2019;42:340–6. http://dx.doi.org/10.1016/j.ctim.2018.12.010	No interesting outcome (emotional aspects)
11	Identification of studies via other methods	Serritella et al. (2021)	Serritella E, Impellizzeri A, Liguori A, Galluccio G. Auriculotherapy used to manage orthodontic pain: a randomized controlled pilot study. Dental Press J Orthod. 2021;26(6):e2119381. http://dx.doi.org/10.1590/2177-6709.26.6.e2119381.oar	LLLT-AT for dental pain
12	Identification of studies via other methods	Velangi et al. (2021)	Velangi CS, Yavagal PC, Nagesh L. Role of auricular laser acupuncture and psychological counseling in reducing nicotine dependence due to smoking: A randomized controlled trial. *Indian J Public Health.* 2021;65(3):243–9. http://dx.doi.org/10.4103/ijph.IJPH_810_20	LLLT-AT for addiction management (smoking cessation)
13	Identification of studies via other methods	Yavagal et al. (2021)	Yavagal PC, L N. Efficacy of Laser Auricular Acupuncture for Smoking Cessation: A randomised controlled trial: A randomised controlled trial. *Sultan Qaboos Univ Med J*. 2021;21(2):e275–81. Available from: http://dx.doi.org/10.18295/squmj.2021.21.02.017	LLLT-AT for addiction management (smoking cessation)
14	Identification of studies via databases and registers	Sari et al. (2022)	Sari MK, Hidayatullah MR. Pengaruh laser acupoint terhadap ansietas pada lansia. *Jurnal Kesehatan Medika Udayana*. 2022;6(2):103–10. http://dx.doi.org/10.47859/jmu.v6i2.164	No control group

15	Identification of studies via databases and registers	Vieira et al. (2022)	Vieira A, Sousa P, Moura A, Lopes L, Silva C, Robinson N, et al. The effect of auriculotherapy on situational anxiety trigged by examinations: A randomized pilot trial. *Healthcare (Basel)*. 2022;10(10):1816. http://dx.doi.org/10.3390/healthcare10101816	Other AT modalities (AT with balls)
16	Identification of studies via databases and registers	Menezes et al. (2022)	Menezes F da S, Chaves E de CL, Mantuani APA, Marino L de S, Alcantara MAR, Nassif MS, et al. Effects of low-power laser auriculotherapy on chronic spinal pain: Randomized clinical trial. *Complement Ther Clin Pract*. 2022;48(101578):101578. http://dx.doi.org/10.1016/j.ctcp.2022.10157	No interesting outcome
17	Identification of studies via databases and registers	Yang et al. (2023)	Yang H-H, Chung Y-C, Szeto P-P, Yeh M-L, Lin J-G. Laser acupuncture combined with auricular acupressure improves low-back pain and quality of life in nurses: A randomized controlled trial. *J Integr Med*. 2023;21(1):26–33. http://dx.doi.org/10.1016/j.joim.2022.10.004	No interesting outcome
18	Identification of studies via other methods	Vaira et al. (2024)	Vaira LA, Massaiu A, Massaiu G, Salzano G, Maglitto F, Lechien JR, et al. Efficacy of auriculotherapy in the control of pain, edema, and trismus following surgical extraction of the lower third molars: a split-mouth, randomized, placebo-controlled, and triple-blind study. Oral Maxillofac Surg. 2024;28(1):279–87. http://dx.doi.org/10.1007/s10006-023-01140-y	LLLT-AT for dental pain
19	Identification of studies via databases and registers	Mantuani et al. (2024)	Mantuani APA, Chaves E de CL, Menezes F da S, Oliveira PE de, Moura C de C, Carvalho LC, et al. Laser auriculotherapy associated with cupping therapy in chronic spinal pain: Randomized controlled clinical trial. *J Bodyw Mov Ther*. 2024;37:194–201. http://dx.doi.org/10.1016/j.jbmt.2023.11.020	No interesting outcome
20	Identification of studies via databases and registers	Vogt et al. (2024)	Vogt KM, Khan A, Alter BJ, Emerick TD, Ibinson JW, Wasan AD, et al. Changes in pain, mood, and functional connectivity following cryo-auriculotherapy in adults with chronic refractory low back pain: An open-label preliminary study. *medRxiv*. 2024. http://dx.doi.org/10.1101/2024.05.17.24301837	Other AT modalities (cryogenic AT)
21	Identification of studies via databases and registers	Shurrab et al. (2025)	Shurrab K, Maroof MS. Efficacy of laser acupuncture as a complementary therapeutic for alleviating depression, anxiety, and stress. Proc Natl Acad Sci India Sect B Biol Sci. 2025; http://dx.doi.org/10.1007/s40011-025-01688-7	Laser acupuncture
22	Identification of studies via databases and registers	Silva et al. (2025)	Silva LR, Toledo LV, Siman AG, Miranda TPS, Santana MCV, Rodrigues YM, et al. Effectiveness of auricular laser acupuncture on anxiety, stress, sleep quality, fatigue and muscle tension of Psychosocial Care Center professionals: a quasi-experimental pilot study. Rev Bras Enferm. 2025;78(3):e20240534. http://dx.doi.org/10.1590/0034-7167-2024-0534	No control group

Supplementary Table S3. Methodological quality of included studies assessed using the PEDro scale

Author (year) Country	PEDro scale criteria											
	Criteria 1*	Criteria 2	Criteria 3	Criteria 4	Criteria 5	Criteria 6	Criteria 7	Criteria 8	Criteria 9	Criteria 10	Criteria 11	PEDro score
Fernandes et al. (2023)+ Brazil [24]	Yes	Yes	No	Yes	No	No	No	Yes	No	Yes	Yes	5
Marques et al. (2023)+ Brazil [25]	Yes	Yes	No	Yes	Yes	No	Yes	Yes	No	Yes	Yes	7
Lin et al. (2024)+ USA [46]	Yes	Yes	Yes	Yes	Yes	No	No	Yes	No	Yes	Yes	6
Lemos et al. (2024)+ Brazil [47]	Yes	Yes	Yes	Yes	Yes	No	No	No	Yes	Yes	Yes	7
Marcondes et al. (2025)+ Brazil [48]	Yes	Yes	Yes	Yes	Yes	No	No	Yes	Yes	Yes	Yes	8
De la Barra et al. (2026)+ Chile [49]	Yes	Yes	Yes	Yes	Yes	Yes	No	Yes	Yes	Yes	Yes	9

PEDro (Physiotherapy Evidence Database) scale criteria:

(1) Eligibility criteria were specified; (2) Random allocation; (3) Concealed allocation; (4) Baseline comparability; (5) Blind subjects; (6) Blind therapists; (7) Blind assessors; (8) Adequate follow-up; (9) Intention-to-treat analysis; (10) Between-group comparisons; (11) Point estimates and variability

*Eligibility criteria item does not contribute to the total score

+Score determined by researchers (Not available in PEDro database)
